# The meaning of dying and death for children, their carers, and families: a scoping review

**DOI:** 10.1186/s12904-023-01295-1

**Published:** 2023-12-04

**Authors:** Christina M. Lamb, Kianna Ramer, Oluwakemi Amodu, Kelsey Groenenboom

**Affiliations:** 1 St. Michael’s College, University of Toronto, Toronto, ON Canada 81 St. Mary Street, M5S 1J4; 2 Athabasca University, Athabasca, AB Canada; 3 Stollery Children’s Hospital, Edmonton, AB Canada; 4 University of Alberta, Edmonton, AB Canada; 5 Foothills Medical Centre, Calgary, AB Canada

**Keywords:** Pediatrics, Palliative care, Family, Caregivers, Scoping review, Death

## Abstract

**Background:**

The meaning of dying and death are underexplored concepts for Canadian children. Subsequently, it is unclear how children and stakeholders make meaning of children’s holistic health needs at the end of life.

**Methods:**

A scoping review of the international scholarly literature was conducted. Thirteen data sources were searched to search the scholarly literature without date limits until January 2022. Studies were included on the basis of population: children (aged 0–19 years), families and caregivers; setting (in Canada and end-of-life or dying phases of living) and concepts of interest (dying and death).

**Results:**

Of the 7377 studies identified, 12 were included for data extraction and content thematic analysis. The themes and subthemes include: 1) valuing the whole person; 2) living while dying; 3) authentic death talk; 4) a supportive approach (with lack and presence of support as subthemes); and, 5) a personalist approach.

**Conclusions:**

There is a pressing need for research into the meaning of dying and death for children, their carers and families in Canada. Lack of holistic care, authentic death talk, specialized pediatric palliative care providers, a personalist approach and communities of support present major gaps in care for Canadian children. Research is urgently needed to address these knowledge gaps to generate policy and support practice for dying children in Canada.

**Supplementary Information:**

The online version contains supplementary material available at 10.1186/s12904-023-01295-1.

## Introduction

Dying is an essential part of living, and death is a significant life event that may occur in the context of healthcare. However, in today’s healthcare, dying and death have, at times, become overmedicalized life experiences [1]. Despite this overmedicalization, a critical gap exists in end-of-life healthcare contexts. Globally, there is a lack of primary pediatric palliative care and specialized pediatric palliative care (SPPC) for dying children [2, 3]. While pediatric palliative care (PPC) applies to the pediatric patient’s entire illness trajectory, palliative care at the end of life is essential for dying children. This is because palliative care approaches appreciate that children are spiritual, psychological and physical persons in their illness, as well as their dying and death experiences [2, 3]. Specialized pediatric palliative care (SPPC) consists of interdisciplinary teams of healthcare professionals who work in dedicated PPC settings to provide care to families with children who have complex needs [4].

However, access to healthcare is often contingent upon available care options. In Canada, SPPC is growing in availability, which is important for providing optimal, ethical and effective care to children dying from terminal diseases and life-limiting conditions [5]. Despite this growth, SPPC is not nationally, equitably available to Canadian children. While Canadian healthcare systems operate on a model of universal access, studies in the Canadian context show that only 18.6% of deceased children who might have benefitted from SPPC received such care (and a quarter received care for less than 8 days) [6]. Furthermore, researchers found that most Canadian children aged 19 and younger (an estimated 81%) who could benefit from SPPC are not receiving it [6].

While addressing the SPPC access gap is critical for children who need it, the paucity of SPPC in Canada uncovers the need to examine other, essential care questions about pediatric dying and death in this country. Missing from the end-of-life literature is a description of what death means for children who die in healthcare contexts. Specifically, in the absence or presence of PPC at the end of life, what is it like for Canadian children to die in Canadian healthcare? What meaning do dying and death hold for Canadian children and their caregivers? While there is little evidence involving children’s perspectives, meaning can be found amid lived, human experiences over significant life events [7, 8]. Therefore, it is essential to ascertain what dying and death mean to dying children, their families and carers in Canada as voiced by these populations themselves [7–11].

To start to elicit a comprehensive understanding of how Canadian children, their families and caregivers make meaning of dying and death, we conducted a scoping review of the Canadian interdisciplinary, scholarly literature. While this review was conducted in the Canadian context, the findings are relevant to other countries since the need for PPC is a global health concern [2, 3]. Our aim was to describe the state of the evidence on the meaning that dying and death hold for children, their families and carers at the end of life. To guide our review, we asked the following research questions: (1) What is known in the scholarly Canadian literature about the meaning that death, dying and end of life hold for Canadian children, their families and caregivers? (2) What interventions exist to support these populations in making meaning of dying and death? (3) What research is needed to bridge research gaps in the Canadian health care system to inform policy and practice on end-of-life care for children and their families?

## Methods

### Study design

This study was guided by Arksey and O’Malley’s methodology along with Levac, Colquhoun and O’Brien’s revisions to their approach [12, 13]. This methodology was appropriate since scoping reviews are conducted to identify complex concepts over which there is only emerging literature [12]. Scoping reviews are also undertaken to generate future studies that will address existing gaps identified in the scholarly literature, which further aligns with our research questions [12, 13]. Following Arksey and O’Malley’s approach, we conducted our review by: (1) identifying the research question(s); (2) identifying relevant studies; (3) selecting the studies; (4) charting the extracted data, and (5) summarizing and reporting the results [12]. Additionally, we used the Preferred Reporting Items for Systematic Reviews and Meta-Analyses extension for Scoping Reviews and guidance from the Joanna Briggs Institute Manual for Evidence Synthesis to inform our search strategy and review of the literature [14, 15].

### Search strategy

After identifying our research questions in stage 1, the search strategy was developed by a health sciences librarian (LS) in consultation with the principal investigator (PI) for stage 2. A second health sciences library (MK) conducted two updated searches of the literature in 2020 and 2022. LS was responsible for translating the initial search strategy across the databases. The literature search was conducted in August 2019 across 13 databases (see Table 1). Search updates were performed in November 2020 and January 2022 (see Supplementary File B for the full search strategy).

**Table 1:** Databases searched

Medline (All) via OVID (1946—Present)
EMBASE via OVID (1974—Present)
PsycINFO via OVID (1806—Present)
Cumulative Index to Nursing and Allied Health Literature (CINAHL) via EBSCOhost (1936—Present)
Child Development & Adolescent Studies via EBSCOhost (Inception—Present)
Religion & Philosophy Collection via EBSCOhost (1911—Present)
Philosophers' Index via EBSCOhost (1743—Present)
SocIndex via EBSCOhost (1895—Present)
Health Source Nursing, Academic Edition via EBSCOhost (Inception—Present)
Scopus via Elsevier (1976—Present)
Web of Science Core Collection: Citation Indexes via Clarivate (1900—Present)
Dissertations & Theses Global via ProQuest (1861—Present)
Sociological Abstracts (1952—Present)

### Eligibility criteria

The included articles were in English. The populations included in the review were children aged 0–19. Neonates less than one month of age or in neonatal intensive care units were excluded as the amount of data available from a preliminary search of the literature indicated that this population warranted their own review. Articles were further included if they consisted of empirical studies or theoretical papers that provided the perspectives of Canadian children, their families and caregivers on the meaning that dying and death hold for them either directly or as mentioned in relation to end of life and palliative care. Review articles were included if they related to the research questions. Since scoping reviews are iterative by design, we decided by consensus during the review process to include international articles if they bridged specifically with a Canadian context and otherwise met all other inclusion criteria. Given the paucity of literature in this area, we subsequently included one study that spanned Canada and the United States and a review paper that addressed international literature inclusive of Canadian studies.

Articles were excluded if they focused on sudden or accidental deaths or traumas that immediately caused death. Since our research questions focus on the meaning of dying and death as voiced by children, conversations would be necessary with children before they died.

### Article selection

**Fig. 1:**
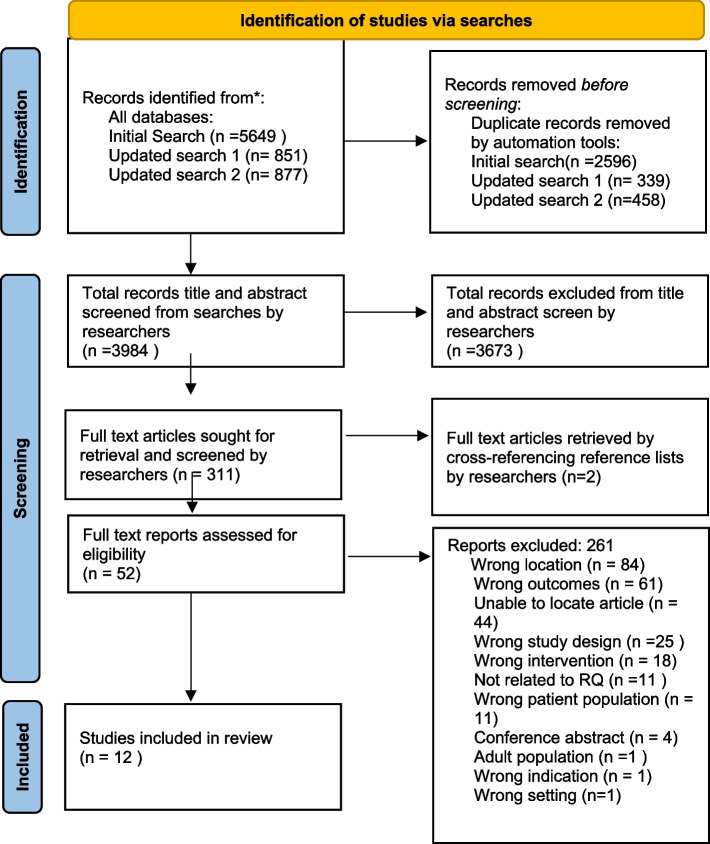
Identification of studies via searches From:  Page MJ, McKenzie JE, Bossuyt PM, Boutron I, Hoffmann TC, Mulrow CD, et al. The PRISMA 2020 statement: an updated guideline for reporting systematic reviews. BMJ 2021;372:n71. doi: 10.1136/bmj.n71

At least 2 researchers independently conducted a two-step screening process in stage 3 of all articles across all searches which were uploaded and deduplicated by the research librarians into Covidence [16]. The screening process involved a minimum of 2 researchers examining the remaining articles by title and abstract according to the research questions and eligibility criteria. Eligible articles then underwent full-text screening prior to undergoing data extraction. Reference lists of included articles were independently screened by 2 researchers, and further articles were subsequently included if they were eligible [12]. The research team communicated at the beginning, halfway point, and end of the screening processes to discuss article assessment [13]. Any conflicts in the screening process were resolved through discussion, consensus or by the PI. Article screening, inclusion and exclusion with rationale were captured with a Preferred Reporting Items for Systematic Reviews and Metanalysis flowchart (see Fig. 1) [17].

### Data extraction and analysis

In stage 4, data were extracted into Excel spreadsheets by a minimum of two, independent researchers. Headings that data were extracted under coincided with the research questions and consisted of the author, region, and country; purpose or research questions; methodology and methods; how the meaning of death and dying was captured and the major findings as well as limitations of each article. Since scoping reviews do not seek to assess the quality of evidence or the bias associated within and across each study, we did not conduct quality or risk of bias assessments in our review [12, 14]. The results were collated, summarized and reported in stage 5 by tabulating the data to present a numerical account of the number, type and distribution of studies included in our review according to the headings we used to map and extract the data (Table 2) [12, 13].

**Table 2:** Data Extraction Chart

Author/Year/Region	Purpose or Research Questions	Methodology and Methods	Population: Meaning of Death and Dying	Major Findings	Major Limitations
Davies, B., Steele, R., Collins, J.B., Cook, K. & Smith, S. (2004)—Vancouver, British Columbia, Canada [18]	To evaluate the impact of a respite program at Canuck Place Hospice in its first 30 months of operation	PAR, mixed-methods, phased study. Face-to-face interviews with 18 families lead to the development of a mail- out survey sent to 144 families. 65 parents completed all or part of the Respite Care Questionnaire for parents	Population: parent’s perspectives Describes meaning that respite care in Canuck Place gave to the families’ experiences with a dying child, including practical relief, opportunities for dying child to talk about death, getting support from families in similar situations	Respite at Canuck Place provided families with needed relief from the emotional and physical burdens of caregiving, a sense of belonging, activities which promote a focus on living rather than dying, opportunities for children, siblings, and parents to have safe conversations about death and dying with skilled staff who serve as patient advocates and mediators to the family. Exposure to death at Canuck Place was difficult for families, but prepared them for what was to come and even provided examples of death that can be positive and not traumatic for the children	Limited access to and development of respite care for families experiencing a pediatric death; survey focused on program evaluation rather than how program specifically impacted meaning of death and dying
Davies, B., Collins, J. B., Steele, R., Cook, K. et al. (2005)—British Columbia, Canada [19]	To investigate the experiences of dying children and their siblings at the Canuck Place, pediatric hospice in Vancouver	Mail-out questionnaires completed and included by 25 ill children and 41 siblings. Face-to-face interviews with 4 ill children and 10 siblings	Population: children (ill children and their siblings) Describes satisfaction and meaning of hospice interventions and experiences for both dying children and their siblings	Nearly all children and siblings reported having a positive experience with the program's activities and environment. Specific valued experiences included the feeling of being understood and the presence of caring staff, a sense of identity/belonging in the shared experience with other ill children/families, fostered independence for sick children and respite for family members, and a special environment which allowed children to concentrate on living, not dying. Suggestions for improvement mainly focused on expanding activities and services for adolescents	Limitations of surveying children included the exclusion of children whose disabilities made them unable to participate in verbal interviews or written surveys and children
Nicholas,D. B., Barrera M., Granek L., D'Agostino N. M., Shaheed J., Beaune L., Bouffet E., & Antle B. (2017) – Canada [20]	To explore the concept of spirituality/hope in parents of children with cancer facing a poor prognosis in a pediatric hospital in a large multicultural city	Longitudinal grounded theory; semi-structured interviews addressing meaning of hope/change of hope over time of 35 parents (26 mothers, 9 fathers) within 3 months of child’s diagnosis; 30 parents at 6 months of diagnosis, and 27 parents at 9 months of diagnosis	Population: parents Themes of how parental spirituality in the context of having children with a poor prognosis of cancer and the transcendent meaning that spirituality brought to their experiences: spirituality and hope; roles of spirituality; illness-related changes in spirituality and support from a faith community	For some parents, personal spirituality gave a context of meaning to their family's experience of suffering, leading to a sense of comfort, solace, and grounding. Parents found belief in a higher power to relieve their own sense of helplessness/lack of control. Some parents reported spirituality giving meaning and comfort to both child and parent when facing the prospect of dying and reoriented their feelings of fear and grief. Parents reported spirituality mainly remained stable during the course of the study, although sometimes it changed in response to circumstances	Sample included families from only one Canadian pediatric oncology unit and represented parents with predominately Christian spirituality. Study did not address children's concept of spirituality in relation to making meaning of the end of life
Russel C.E., Widger K., Beaune L., Neville A., Cadell S., Steele R., Rapoport A., Rugg M. & Barrera M. (2018) -Canada [21]	To explore the impact of a dying sibling on healthy children, focusing on their involvement in the dying child's life and the impact of their involvement on their grief, coping, and life outside of family	Prospective, longitudinal, qualitative study utilizing interpretive descriptive methodology employing semi-structured interviews of 10 siblings of dying children over 2 years	Population: siblings Captures perspectives of how healthy siblings make meaning of the dying process of their sibling- some found the experience strengthening for their family while others found it isolating; coping strategies included quality time with the ill child, taking on a caregiving role, spirituality and music. This article revealed that communication about death between the family/parents and siblings was often lacking and siblings struggled to express how they could be supported in their grief, concluding that siblings need increased support from parents and healthcare professionals in how they make meaning of their sibling’s death	Findings of this study include the unique roles, communication experiences, and needs of siblings of dying children	Sample population derived from a single tertiary facility, small sample size
Flavelle S. (2011)—Halifax, Nova Scotia, Canada [22]	Thematic summary of an adolescent's journal capturing his experience with cancer 3 months prior to his death	Case study utilizing phenomenological analysis of a dying youth’s journal	Population: children Explores the lived experience of a 15-year-old facing death from cancer in the pediatric palliative care context. Themes of his journal entries include how he used humor to make meaning of death and dying; viewing dying from an adolescent developmental perspective; considering the impact of his dying on his family relationships, and his journey with spirituality and hope to bring meaning to and during his illness	Listening to the voiced experience of a dying adolescent facing death can help researchers and clinicians understand how an adolescent made meaning of the dying process. Study sheds light on how this life experience may be meaningful to similar persons and further appreciate what it is like to be an adolescent dying from cancer	Single case study report; further research into how other adolescents view their own end of life experience needed
deJong, M. & Kane L. (2006)—Edmonton, Alberta, Canada [23]	To evaluate parental satisfaction with a bereavement program at the Stollery Children's Hospital	Mailed-out, mixed-methods (open and scaled questions) questionnaires completed by 21 of 81 eligible, bereaved families. Questionnaire was adapted from "Whispers of Hope" bereavement program evaluation survey from Duke University. The questionnaire was revised and adapted to local context with expert validation post interviews with Stollery staff and a parent	Population: parents of families of bereaved children The meaning families made of the dying and death of their ill child were captured by themes in the open-ended questions/additional comments: extending care; knowing someone cared and learning from grief	Bereavement programs are critical to families experiencing a pediatric death. Staff involved in Pediatric Palliative Care can become an intimate part of the families experience and grief, and maintaining contact with families after the death of the child can reduce feelings of abandonment/isolation and help families feel supported	Low response rate to survey (25.6%). Many bereaved families did not live in the city where their child died in hospital and could not participate in all aspects of the bereavement follow up program (i.e. memorial service)
Steele R. (2005) – Canada [24]	To explore the experience of parents with children dying from a neurodegenerative, life-threatening illness (NLTIs)	Grounded theory study with interviews/observations of 8 families (29 family members, 10 sick children- two families had two sick children with NLTIs)	Population: family members (parents) Describes strategies families used to make meaning of their child's terminal illness and the dying process	Strategies to find meaning in child's illness and dying included taking each day as it came, finding the positives/opportunities for growth in their situation, wrestling with existential questions of spirituality and sickness (some parents found peace in God to manage their situation, others lost faith or became bitter towards God), and participating in research with the hope of helping other families and children in the future	Small sample size, potentially lacking in cultural diversity
Peterson C. L. (2013). Includes Canada [25]	To report a concept analysis of spiritual care for children at the end of life	Literature search of databases CINAHL, ATLA and PubMed, inclusion of studies published up to the end of 2012, English, peer-reviewed articles yielded result of 51 included studies. Each article was reviewed for attributes of spiritual care, which were categorized into: attributes; antecedents; consequences; surrogate terms, and related concepts for analysis completed by Rodger’s evolutionary method	Population: children Discusses how attributes of spiritual care in the pediatric palliative population can assist the child to find meaning during the dying process	Assisting a dying child with cancer to express their feelings, beliefs, and hopes surrounding their death, exploring legacy-making activities, and the use of life stories, faith practices, and altruistic acts (such as cancer research fund-raising) can help some children find spiritual meaning amidst their tragedy	This analysis did not account for pediatric developmental differences in spiritual care needs and did not examine spiritual care interventions related to specific cultural or religious beliefs
Zelcer S., Cataudella D., Cairney A. E.L., Bannister, S. L. (2010) – London, Ontario, Canada [26]	To explore the end of life experience of children with brain tumors and their families	Qualitative study utilizing thematic analysis on data collected through semi-structured focus group interviews of 25 parents of 17 deceased children between 3–12 years post death	Population: parents Maintaining normality, finding hope and strength from their child's resiliency, and conversations about death and dying in the family point to how families found meaning in the dying child process	Themes from family interviews include: the dying trajectory; parental struggles and dying at home. Thematic fundings discuss the desire to maintain normalcy as much as possible and focus on living rather than dying (such as encouraging child continuation in schooling, celebrating accomplishments, and peer friendships and support). Parents reported finding strength by maintaining hope and through the resiliency of their suffering child. Parents also suggested that their child was aware of their own impending death and lead their parents through the dying process and saying goodbye, indicating that children wish to discuss death with their loved ones	Small sample size; interviews with families occurred 3–12 years post child death which may not accurately reflect the actual dying process owing to the lapse of time
Muskat B., Greenblatt A., Anthony S., Beaune L., Hubley P., Newman C., Brownstone D., & Rapoport A. (2020)—Ontario, Canada [27]	To explore the experiences and coping strategies of physicians, nurses, and social workers working with dying children	Qualitative study utilizing an interpretive descriptive approach; face-to-face individual, semi-structured interviews with 25 healthcare professionals (physicians n = 8, nurses n = 8, social workers n = 9) who regularly cared for dying children in an acute-care setting	Population: carers Discusses both the difficulties and the meaningful aspects of providing end of life care to pediatric patients including giving healthcare providers a sense of reward; meaning in providing the best care possible to children and developing greater personal appreciation and understanding of what makes life meaningful	Participants reflected on how they make meaning of the death of pediatric patients in terms of the privilege of being with their patients at the end of life. Meaning was captured in their facilitating high quality palliative care, and even exploring their own personal perspectives of the meaning of life. Healthcare providers also shared that their own personal beliefs on death and achieving a sense of peace and acceptance in regards to the dying process resulted in their greater ability to cope	Sample does not represent the perspectives of new staff experiences, nor does the study examine the difference between specialties
Champagne, E. (2008)—Quebec, Canada [28]	To describe children's expressions of living and dying in the context of an interactive Christian programme in Quebec and provide a theological/social interpretation of their expressions / experiences of death	Discussion article on the author's use of natural observation and interpretations of three groups of child participants who voiced understandings of death in a voluntary, theological exercises program in Quebec	Population: children Children's participation in this religious formation program gave space for their expression of what death and dying means to them. Children expressed ideas of suffering and loss, the universality of death, questions of where people go when they die, and a practice of speaking with the deceased "in their hearts"	Children actively and spontaneously engage in discussions on death and dying, and listening to their own insights and experiences can help adults appreciate children’s voices and spiritual understandings of death. Children’s understanding of death and dying will vary based on their psychosocial development and experiences, but the utilization of stories and symbols may aid children in voicing their inner understandings to the outside world. Socio-political contexts can restrict children’s faith expression and search for transcendent meaning about death. Religious freedom should be encouraged in Canada to inform children and parents/ families worldviews	The observations made in this article were from a small sample size at one religious formation program in Quebec. Further research to children's voices and worldviews on death and dying are needing with larger populations and in more diverse, cultural contexts
Gagnon, M., Kunyk, D. (2021). Canada (data from 6 PICUs across Canada) [29]	To analyze nurses’ experiences of moral distress working with children who die in Canadian PICUs	Secondary, qualitative content analysis conducted on data from primary interviews with nurses in a parent study exploring the moral distress in Canadian PICUs. Transcripts were selected from parent study that focused on nurses’ perspectives. Inductive analysis generated new lines of inquiry to respond to secondary research questions on how nurses in parent study made meaning of moral distress related to their experiences of working with dying children who died in PICU contexts	Population: carers (nurses) A child’s dying from the perspective of nurses should be dignified; dignity was captured as negatively meaningful: a child’s death in the PICU ought not be a salvage experience; children’s dying in PICU settings was often drawn out, painful and worthy of better ethical decision making regarding moving from disproportionate to proportionate care. Lack of ethical knowledge inhibits, stalls or stagnates the ethical care of dying children which inhibits healthcare professionals’ responsibilities to the dying child. Focusing on extrinsic elements i.e. technology distracts from the personal; nurses need to move beyond internal constraints to do what they ought to do for the dying child patient as opposed to focusing on what they can’t do owing to external constraints	Three themes developed from the content analysis: 1) a dignified death and balancing best interests; 2) burden of insider knowledge, and 3) constraints to nursing roles and responsibilities. Dying and death for children need to be dignified which is not conditional or controlled; it is about capturing the humanity and personhood of the child who is dying and ensuring that extraneous factors such as technology, lack of ethics knowledge and power imbalances in healthcare professional relationships do not overshadow the experience of a child’s death. Palliative care needs to be moved into the PICU context so that children can die well. Dying needs to be normalized in a responsible way; extraordinary/ disproportionate treatment should not take away from the ordinary yet extraordinary care and meaning of each child’s life, albeit a short life	Data from parent study were obtained a decade ago; however, findings still relevant in light of advancing technologies to prolong life for children in and out of critical and intensive care settings. Some of these advances are not proportionate to the context of pediatric dying and death. Medical advances in treatment not being met with simultaneous growth in specialized pediatric palliative care at the end-of-life in Canada. It is not clear how effective change can occur. Moral distress is yet to be resolved and interprofessional communication has not shown marked improvement to improve nurses’ experiences of working with dying children

Summarizing and reporting the data included organizing the data through content thematic analysis [30]. Content thematic analysis for data analysis was performed using Braun and Clarke’s method, which consists of six phases: 1) immersion in, and familiarization with, the data; 2) generating initial and complete codes; 3) identifying candidate themes; 4) reviewing themes; 5) naming the themes and subthemes (where applicable), and 6) writing the research report [30].

## Results

Across all searches a total of 7377 records were identified through database searching; 3393 duplicate records were removed, leaving 3984 records for title/abstract screening. After the title and abstract screening, 52 articles remained for full text screening of which 12 were eligible for inclusion in the review. Published between 2004 and 2021, the articles consisted of 1 review study, 10 research studies and 1 case study. Five themes and subthemes arose from the analysis consisting of: 1) valuing the whole person; 2) living while dying; 3) authentic death talk; 4) a supportive approach with the subthemes of presence and lack of support; and 5) a personalist approach. The results of the analysis of these studies are captured in the each of these themes and subthemes as laid out in this section.

### Reporting of thematic analysis by themes

#### Theme 1: Valuing the whole person

*Valuing the whole person* was thematically captured across the studies as the understanding that people are psychological, physical, and spiritual persons. The spiritual dimension of being human was particularly relevant across all populations under study. Parents of dying children expressed deep spiritual pain or a strong faith in relation to making meaning of their child’s suffering and death [20]. Parents also reported that a spiritual and moral struggle was necessary for some of them to find meaning in the experience of their dying child [24]. Some parents lost faith; for others it was reaffirmed [20, 24]. Dying children also wrestled with transcendental elements of dying and death; they described their anger toward spirituality or religion in relation to the suffering they were experiencing [22]. Conversely, spirituality was also captured as something that leant meaning to a child’s mortality, even in the midst of their dying [22]. Spirituality and spiritual care can also provide an opportunity for dying children to find meaning in life and suffering [25].

Children and their families reported that some healthcare professionals in hospice settings were able to address the psycho-spiritual dimensions of being a whole person [19, 28]. However, families also found that some healthcare professionals were uncomfortable with discussing spirituality; some healthcare professionals criticized families for considering morality and spirituality as relevant phenomena at the end of life [24]. Other healthcare professionals self-reported the relevance of spirituality as meaningful to the work they did with dying children [27].

#### Theme 2: Living while dying

The theme of *living while dying*revealed that living with a dying child can be all-consuming [24]. The ability for families to access healthcare professionals who have the perspective that dying is part of living alongside the necessary clinical skills to care for dying children is essential [18]. Such skills are a specialized area of care. Having the requisite outlook and skills creates meaningful opportunities for children, their families and carers to be transparent and open about healing, as well as personal and professional growth at the end of life. Utilizing palliative care principles positively emphasizes the life of the child as still living, and does not focus exclusively on their dying [18, 27]. Primary pediatric palliative care and SPPC are therefore relevant for seriously ill children because they support incorporating death into children’s perspectives on life as it is still being lived while allowing them to prepare for their end.

At the same time, *living while dying*is challenging; parents caring for dying children still must live their own lives alongside their dying child. Having a dying child can be paradoxical for parents: knowing that their lives will continue after their child has died [22, 23]. For some siblings of dying children, death elicited fears of the unknown. Specifically, what would death be like for their dying sibling when it happened and for the sibling(s) left behind? [21] Siblings of dying children need support to process their grief after their sibling dies [21]. From the perspective of healthcare professionals, caring for dying children can be demanding, but it also provides time for healthcare professionals to reflect meaningfully on their work with dying children [27]. Children who are *living while dying* in the context of illness may endure pain and suffering [22]. Pain control and interventions to address the multifaceted dimensions of suffering are therefore necessary. However, suffering is not only pain oriented. Supporting dying children to find existential meaning, purpose, and hope amidst dying as part of living is also an essential facet of care for the child who is *living while dying *[25].

#### Theme 3: Authentic death talk

To support children in preparation for death as they live through the dying process, *authentic death talk* is crucial. However, talking about death is not a conversation that all parents feel comfortable or capable of having with their dying child [18, 26]. Some parents, siblings and families of dying children did not discuss the dying or death of a child at all. However, a lack of openness and authentic communication of the reality of what was happening led to some family members feeling “isolated” [21]. However, openly discussing dying and death in some instances brought cohesion, strength and a sense of support to families [21]. Similarly, dying children and youth want to talk about their dying and death and find such conversations important [22, 25, 26]. At times, *authentic death talk* needs to happen on behalf of children who are unable to speak for themselves. Such conversations require skilled, ethical consideration and respect for the need to protect children’s dignity in response to their increased dependency relative to their clinical and holistic care needs [29].

#### Theme 4 and subthemes: a supportive approach (lack or presence of support as subthemes)

Across the studies, the lack and presence of support for dying children are captured through the subthemes of *presence* and *lack of support. The presence of support* refers to families’ need for communities that will help them care for their dying children. Supportive clinical environments such as hospices, palliative care homes or units and bereavement programs can reinforce the importance of a specialized and appropriately contextualized healthcare environment when it is warranted for a child at their end of life [23].

*Lack of support* consists of healthcare professionals who showed a lack of appreciation or respect for the spiritual aspect of being human [24]. Similarly, expertise and training in SPPC as well as ethical preparedness for end-of-life care is lacking in Canada. Such expertise is necessary to provide excellent care to children who are dying [29] *Lack of support* also includes the dearth of practical resources. Some families reported that nothing in their dying child’s care trajectory was easily accessible: equipment, respite, interventions, and how to manage at home [24]. Families also remarked on the absence of community support in the context of Canadian healthcare. As Davies et al. found, “Traditional sources of family support such as extended families, churches and social organizations are no longer as prominent in our increasingly secular society” [18]. In response to the lack of holistic care and community connections, a *supportive approach* to care is needed in Canada that incorporates supportive sources of community into the care of dying children.

#### Theme 5: A personalist approach

Personalism takes the holistic aspects of being human into consideration through the lens of bioethics and by emphasizing the concept of interdependency [31]. While disagreements may arise between family and/or caregivers over what course of action should be taken in the dying child’s care, *a personalist approach* is needed, at times, to refocus such disagreements in an ethical light. For example, Gagnon and Kunyk identified that caregivers and families need to better differentiate when to transition dying children in pediatric intensive care units (PICUs) to SPPC [29]. In some instances, dying children in ICUs do not receive the SPPC they need because of interprofessional disagreements over when to transition them from active treatment to comfort care [29]. Gagnon and Kunyk point out that unresolved or prolonged disagreements can become an ethical concern for the dying child if it extends their suffering [29]. Moreover, Gagnon and Kunyk show that interprofessional disagreements may lead to healthcare professionals’ moral distress, which can manifest in inaction on behalf of the patients. Such inaction can be compounded by a lack of ethics expertise to navigate disagreements over complex care issues for dying children in PICU contexts. *A personalist approach* could be helpful in these situations because it necessitates moral engagement across disciplines, highlights interdisciplinary over disciplinary approaches to ethics, and accepts that healthcare occurs in light of interdependent relationships, which are essential in pediatric care.

## Discussion

### Main findings of the study

The findings of this review reveal that little research has been conducted on the meaning that dying and death hold for children, their families, and carers at their end-of-life as voiced by these populations in the Canadian healthcare context. However, across the studies included in this review, several needs and disparities in end-of-life care for the populations under study were uncovered. Specifically, children and their families voiced the need for PPC at the end of life where their faith will be respected, death will be discussed, dying will be valued as an important phase of life, and where all persons involved in the care of the dying child will be valued as psychological, physical, and spiritual persons [3]. Although PPC approaches at the end of life espouse holistic perspectives toward care, these findings relate the need to attend to whether and how the needs identified in this review are currently being met in the Canadian context as voiced by children, their families and caregivers themselves [11, 32]. Additionally, while pediatric palliative and hospice care are becoming more established in Canada, Widger et al. show that SPPC programs are not yet matching the demand for children in Canada who need them [6]. To address these needs, Canadian children ought to have better access to SPPC when their dying and death warrant this degree of medical attention and to best attend to their holistic health needs at the end of life [11, 31–33]. Studies that explore the meaning of dying and death across primary PPC and SPPC contexts may provide insights to inform policies that will advance more care provision in this area.

The findings of this scoping review reveal that limited interventions exist for the populations under study to express what dying and death meant to them. One study indicated that staff in Canuck Place supported children to make sense of death in ways they would understand, but this process was not described [19]. Subsequently, it is not clear how this support could be interpreted as an intervention. Another study captured ways in which an adolescent used journaling and humor to make sense of his dying process [22]. Other researchers assessed pediatric hospice programmes and centres and what dying means for some children in specific faith-based contexts [18, 19, 23, 28]. Some studies offered insight into how parents and siblings of dying children made meaning of death or dying through *processes* related to these events [24–26]. One study found that carers made meaning of a child’s dying by acknowledging the privilege of being present in the care of dying children and by reflecting on the meaning of life [27]. However, what dying and death are and mean to children, their families and carers in the context of Canadian healthcare as stand-alone phenomena were not well explicated overall.

The lack of interventions uncovered in our scoping review on how to support populations to make meaning of children’s dying and death is an important discovery and reveals the pressing need for research in this area. These needs are ethical. Without the necessary expertise to provide care that will meet the needs of children dying in a healthcare context as voiced by children, their families and carers themselves, the lack of knowledge in this area may become a moral concern. Since cultural and community perceptions of morals and values can drive health care policy and practice, it is crucial to engage with Canadian children and their families and carers to appreciate what these perspectives are by first ascertaining how these populations make meaning of these life events [8, 9, 34].

### What this study adds

To capture this meaning, open discussions are needed to attend to children as psychological, physical, and spiritual persons. While the need for children to have open discussions about death has been echoed by other researchers in the Canadian literature, studies in this review indicate that children’s faith and spiritual values need to be acknowledged in death talk as well [19, 22, 24, 28, 35]. Although Canadian palliative care and medical organizations acknowledge the relevance of these values, the studies in this review indicate that these values are not necessarily or consistently reflected in practice [9, 33]. Incorporating diverse discussions about faith and spirituality into pediatric care could start to address this gap. Furthermore, as the findings indicate, research is needed to better understand what it means to be a dying child within the Canadian healthcare context. For children’s holistic care to be fully articulated, researchers need to consider the meaning of dying and death as voiced by children themselves. Where children cannot voice their needs, due to age or pathophysiological limitations, research is further indicated to explore the meaning of dying in death in tandem with children’s families and caregivers who can lend voice to children’s needs in these situations [7, 11]. Specifically, the research reviewed in our study shows that SPPC is required in some Canadian PICUs to optimize a dying child’s final phase of life [29]. Studies are also needed in PICUs to measure whether incorporating SPPC or transferring patients to SPPC units would holistically improve children’s end-of-life trajectories, experiences and pediatric healthcare practice.

While some policies exist to promote the need for palliative care in Canada, focused attention and ongoing efforts need to be paid to promoting primary PPC and SPPC in this country and internationally [6, 36–38]. Further work is also needed to ascertain the effective entry points of these care profiles into the care trajectories of dying Canadian children [6, 36, 37]. As a next step, qualitative studies employing phenomenological research methods could shed light on the meaning of being a dying Canadian child to articulate what these experiences are like to inform policies and practice.

### Limitations of the study

This study had limitations. While Canada has two official languages, we included English articles in our study because we did not have French-speaking researchers. Future studies exploring the meaning of Canadian pediatric dying and death should include Francophone researchers or translators. Additionally, we limited our search to the scholarly literature. Unpublished, gray literature was not included.

## Conclusions

The lack of evidence around the meaning of dying and death for Canadian children, their families and caregivers poses a challenge to implementing care that uniquely responds to children’s holistic needs at the end of life. Faced with this knowledge gap and the lack of SPPC for Canadian children who need it, research is urgently needed to organize baseline and consistent end-of-life care for dying Canadian children. This study provides an initial step in establishing a national knowledge base for further research, policy and practice into the meaning that dying and death hold for Canadian children, their families, caregivers, and stakeholders invested in essential end-of-life care for some of Canada’s most vulnerable populations.

### Supplementary Information

**Additional file 1.**

**Additional file 2.**

## Data Availability

Not applicable.

## Abbreviations

PI: Principal investigator
HCP: Healthcare professional
NICU: Neonatal intensive care unit
PICU: Pediatric intensive care unit
PPC: Pediatric palliative care
SPPC: Specialized pediatric palliative care
PRISMA: Preferred reporting items for systematic reviews and meta-analyses
PRISMA-ScR: Preferred reporting items for systematic reviews and meta-analyses extension for scoping reviews
