# Supplementary material for: The meaning of dying and death for children, their carers, and families: a scoping review
Source: BMC Palliat Care. 2023 Dec 4;22:194. doi: 10.1186/s12904-023-01295-1 (PMC10694886; doi:10.1186/s12904-023-01295-1)
Supplement: Supplementary file 1 — Additional file 1. [file 12904_2023_1295_MOESM1_ESM.docx]

**Search Methods**

This review was designed and conducted in adherence to the Preferred Reporting ITems for Systematic Reviews and Meta-Analyses extension for Scoping Reviews (PRISMA-ScR) [1], and guidance from the Joanna Briggs Institute Manual for Evidence Synthesis, Chapter 11: Scoping Reviews [2].

The search strategy was developed by an experienced health sciences librarian (LS) in consultation with the research team. LS was responsible for translating the search strategy across the following databases:

- Medline (All) via OVID (1946 - Present)
- EMBASE via OVID (1974 - Present)
- PsycINFO via OVID (1806 - Present)
- Cumulative Index to Nursing and Allied Health Literature (CINAHL) via EBSCOhost (1936 - Present)
- Child Development & Adolescent Studies via EBSCOhost (Inception - Present)
- Religion & Philosophy Collection via EBSCOhost (1911 - Present)
- Philosophers' Index via EBSCOhost (1743 - Present)
- SocIndex via EBSCOhost (1895 - Present)
- Health Source Nursing, Academic Edition via EBSCOhost (Inception - Present)
- Scopus via Elsevier (1976 - Present)
- Web of Science Core Collection: Citation Indexes via Clarivate (1900 - Present)
- Dissertations & Theses Global via ProQuest (1861 - Present)
- Sociological Abstracts (1952 - Present)

The search strategy was derived from three components: 1) terms representing meaning, attitudes, perceptions of death were combined with terms representing the pediatric population and terms representing end-of-life, dying, and terminal care; 2) terms representing meaning, attitudes, perceptions of death were combined with terms representing family and caregivers, including professional caregivers, and terms representing the pediatric population at the end-of-life; 3) terms representing end-of-life care were combined with terms representing the pediatric population and terms intended to retrieve Canadian studies. The search components were searched using a combination of controlled terms (subject headings) wherever they were available and natural language keywords. The results from each search component were combined using the Boolean operator OR to generate a final set of records (see Supplementary File X for full search strategy for each database). In order to increase search sensitivity, no date, language, or format limits were applied.

The search was initially conducted in August, 2019 and two search updates were conducted in November, 2020, and January 2022. The original search strategies unique to each database were re-run without changes and new articles were collected. In total, 7377 records were identified through database searching and 3393 duplicate records were removed leaving 3984 records for title/abstract screening.

Additional publications were retrieved through scanning the reference lists of relevant articles.

Results were managed using the systematic review software, Covidence. This software was used to automatically deduplicate search results upon import and was used to facilitate title/abstract and full-text screening of results.

1. <http://www.prisma-statement.org/Extensions/ScopingReviews>
2. <https://wiki.jbi.global/display/MANUAL/Chapter+11%3A+Scoping+reviews>

**Supplementary File B - Search Strategies by Database**

**Ovid MEDLINE(R) and Epub Ahead of Print, In-Process & Other Non-Indexed Citations and Daily <1946 to November 4 2020>**

--------------------------------------------------------------------------------

1 Attitude to death/ or death/ or (death or dying or "end of life" or euthanasia or ((assisted or assistance) adj3 (death or dying)) or MAID or palliative care or terminal care).ti,kf. (185795)

2 comprehension/ or concept formation/ or perception/ or (meaning or attitude* or perception* or belief* or perspective* or concept* or understand* or comprehen*).ti,kf. (483649)

3 1 and 2 (7256)

4 Psychology, child/ or child development/ or (child* or teen* or adolescen* or p?ediatric* or minor*1 or neonat* or newborn* or infan*).ti,kf,jw,nw. (1745536)

5 3 and 4 (795)

6 ((meaning or attitude* or perception* or belief* or perspective* or concept* or understand* or comprehen*) and (death or dying or "end of life" or euthanasia or ((assisted or assistance) adj3 (death or dying)) or MAID or palliative care or terminal*) and (child* or teen* or adolescen* or p?ediatric* or minor*1 or neonat* or newborn* or infan*)).ti,kf. (599)

7 (((child* or adolescen* or teen* or p?ediatric* or minor*1) adj4 (meaning or attitude* or perception* or belief* or perspective* or concept* or understand* or comprehen*)) and ((meaning or attitude* or perception* or belief* or perspective* or concept* or understand* or comprehen* or fear or anxiety or anxious or scared) adj4 (death or dying or afterlife or heaven or "end of life" or euthanasia or ((assisted or assistance) adj3 (death or dying)) or MAID or palliative care or terminal care))).ti,ab,kf. (299)

8 death/px and (child* or teen* or adolescen* or p?ediatric* or minor*1 or infan* or neonat* or newborn* or minor*).mp. (5)

9 5 or 6 or 7 or 8 (940)

10 palliative care/ or exp terminal care/ or ("end of life" or palliative care or terminal* or hospice* or incurable or impending death or life limiting or life shortening or (dying adj3 (child* or adolescen* or teen* or minor*1 or infan* or neonat* or newborn*))).ti,ab,kf. (562560)

11 9 and 10 (396)

12 exp Family/ or exp Caregivers/ or exp "Attitude of Health Personnel"/ or (family* or families* or parent* or brother* or sister* or sibling* or caregiver* or nurse* or physician* or oncologist* or cardiologist* or ((health* or home care) adj (worker* or professional*))).ti,kf. (881763)

13 3 and 12 (3080)

14 ((family* or families* or parent* or brother* or sister* or sibling* or caregiver* or nurse* or physician* or oncologist* or cardiologist* or ((health* or home care) adj (worker* or professional*))) adj4 (meaning or attitude* or perception* or belief* or perspective* or concept* or understand* or comprehen*)).ti,ab,kf. (67899)

15 ((meaning or attitude* or perception* or belief* or perspective* or concept* or understand* or comprehen*) adj4 (death or dying or "end of life" or euthanasia or ((assisted or assistance) adj3 (death or dying)) or MAID or palliative care or terminal care or terminal* ill*)).ti,ab,kf. (8181)

16 14 and 15 (1236)

17 13 or 16 (3613)

18 ((child* or teen* or adolescen* or p?ediatric* or infan* or neonat* or newborn*) and (death or dying or terminal* ill* or end of life or incurable or life limiting)).ti,kf. (14005)

19 ((child* or teen* or adolescen* or p?ediatric* or infan* or neonat* or newborn*) adj3 (death or dying or terminal* ill* or end of life or incurable or life limiting)).ab. (20691)

20 18 or 19 (29925)

21 17 and 20 (395)

22 *palliative care/ or exp *terminal care/ or ("end of life" or palliative care or terminal care or terminal* ill* or hospice*).ti,ab,kf. (86014)

23 (canad* or british columbia or alberta or saskatchewan or manitoba or ontario or quebec or new brunswick or nouveau brunswick or nova scotia or prince edward island or newfoundland or labrador or nunavut or nwt or northwest territories or yukon).af. (1114868)

24 22 and 23 (5977)

25 adolescent health services/ or child health services/ or (child* or teen* or adolescen* or p?ediatric* or infan* or neonat* or newborn*).ti,ab,kf,jw,nw. (2368198)

26 24 and 25 (485)

27 11 or 21 or 26 (1059)

28 animals/ not humans/ (4575628)

29 27 not 28 (1194)

**Ovid Embase <1974 to 2020 November 4>**

--------------------------------------------------------------------------------

1 attitude to death/ or death/ or dying/ or (death or dying or "end of life" or euthanasia or ((assisted or assistance) adj3 (death or dying)) or MAID or palliative care or terminal care).ti,kw. (464317)

2 perception/ or conception/ or awareness/ or comprehension/ or (meaning or attitude* or perception* or belief* or perspective* or concept* or understand* or comprehen* or fear or anxiety or anxious or scared).ti,kw. (781311)

3 1 and 2 (15512)

4 concepts of death/ (1)

5 3 or 4 (15512)

6 child development/ or child psychology/ or (child* or teen* or adolescen* or p?ediatric* or minor*1 or neonat* or newborn* or infan*).ti,kw,jx. (1989673)

7 5 and 6 (1521)

8 (((child* or teen* or adolescen* or p?ediatric* or infan* or neonat* or newborn*) adj4 (meaning or attitude* or perception* or belief* or perspective* or concept* or understand* or comprehen*)) and ((meaning or attitude* or perception* or belief* or perspective* or concept* or understand* or comprehen*) adj4 (death or dying or "end of life" or euthanasia or ((assisted or assistance) adj3 (death or dying)) or MAID or palliative care or terminal care or terminal* ill*))).mp. (546)

9 7 or 8 (1771)

10 exp terminal care/ or exp palliative therapy/ or ("end of life" or palliative care or terminal* or hospice* or incurable or end stage or life limiting or life shortening or impending death or active* dying or (dying adj3 (child* or teen* or adolescen* or p?ediatric* or infan* or neonat* or newborn*))).ti,ab,kw. (775991)

11 9 and 10 (679)

12 exp family attitude/ or family/ or exp family decision making/ or exp family coping/ or caregiver/ or exp health care personnel/ or (family* or families* or parent* or brother* or sister* or sibling* or caregiver* or nurse* or physician* or oncologist* or cardiologist* or ((health* or home care) adj (worker* or professional*))).ti,kw. (1954932)

13 1 and 2 and 12 (6517)

14 ((family* or families* or parent* or brother* or sister* or sibling* or caregiver* or nurse* or physician* or oncologist* or cardiologist* or ((health* or home care) adj (worker* or professional*))) adj4 (meaning or attitude* or perception* or belief* or perspective* or concept* or understand* or comprehen*)).ti,ab,kw. (83827)

15 ((meaning or attitude* or perception* or belief* or perspective* or concept* or understand* or comprehen*) adj4 (death or dying or "end of life" or euthanasia or ((assisted or assistance) adj3 (death or dying)) or MAID or palliative care or terminal care or terminal* ill*)).ti,ab,kw. (11056)

16 14 and 15 (1708)

17 13 or 16 (7221)

18 ((child* or teen* or adolescen* or p?ediatric* or infan* or neonat* or newborn* or minor*1) and (death or dying or terminal* ill* or end of life or incurable or life limiting)).ti. (11646)

19 ((child* or teen* or adolescen* or p?ediatric* or infan* or neonat* or newborn*) adj3 (death or dying or terminal* ill* or end of life or incurable or life limiting)).mp. (44444)

20 18 or 19 (46359)

21 17 and 20 (541)

22 exp *terminal care/ or exp *palliative therapy/ or ("end of life" or palliative care or terminal care or terminal* ill* or hospice*).ti,ab,kw. (109810)

23 (canad* or british columbia or alberta or saskatchewan or manitoba or ontario or quebec or new brunswick or nouveau brunswick or nova scotia or prince edward island or newfoundland or labrador or nunavut or nwt or northwest territories or yukon).af. (1589792)

24 22 and 23 (9611)

25 exp child health care/ or (child* or teen* or adolescen* or p?ediatric* or infan* or neonat* or newborn*).ti,ab,kw,jx. (2826195)

26 24 and 25 (927)

27 11 or 21 or 26 (1803)

28 limit 27 to (conference abstract or "conference review") (581)

29 27 not 28 (1222)

30 animal/ not human/ (1044413)

31 29 not 30 (1396)

**Ovid PsycINFO <1806 to November Week 1 2020>**

--------------------------------------------------------------------------------

1 exp "Death and Dying"/ or exp Death Attitudes/ or exp Death Anxiety/ or (death or dying or "end of life" or euthanasia or ((assisted or assistance) adj3 (death or dying)) or MAID or palliative care or terminal care).ti,id. (58753)

2 meaning/ or meaningfulness/ or comprehension/ or concept formation/ or (meaning or attitude* or perception* or belief* or perspective* or concept* or understand* or comprehen*).ti,id. (570672)

3 childhood development/ or adolescent development/ or (child* or teen* or adolescen* or p?ediatric* or minor*1 or infan* or neonat* or newborn*).ti,id,jx. (668257)

4 1 and 2 and 3 (1139)

5 (((child* or adolescen* or teen* or p?ediatric* or infan* or neonat* or newborn*) adj4 (meaning or attitude* or perception* or belief* or perspective* or concept* or understand* or comprehen*)) and ((meaning or attitude* or perception* or belief* or perspective* or concept* or understand* or comprehen*) adj4 (death or dying or "end of life" or euthanasia or ((assisted or assistance) adj3 (death or dying)) or MAID or palliative care or terminal care or terminal* ill*))).ti,ab,id. (604)

6 4 or 5 (1326)

7 exp palliative care/ or terminally ill patients/ or hospice/ or ("end of life" or palliative care or terminal* or hospice* or incurable or life limiting or life shortening or impending death or active* dying or (dying adj3 (child* or adolescen* or teen* or infan* or neonat* or newborn* or minor*1))).ti,ab,id. (43250)

8 6 and 7 (283)

9 Family/ or exp Family Members/ or caregivers/ or exp health personnel attitudes/ or (family* or families* or parent* or brother* or sister* or sibling* or caregiver* or nurse* or physician* or oncologist* or cardiologist* or ((health* or home care) adj (worker* or professional*))).ti,id. (409102)

10 2 and 9 (69711)

11 ((family* or families* or parent* or brother* or sister* or sibling* or caregiver* or nurse* or physician* or oncologist* or cardiologist* or ((health* or home care) adj (worker* or professional*))) adj4 (meaning or attitude* or perception* or belief* or perspective* or concept* or understand* or comprehen*)).ti,ab,id. (67668)

12 10 or 11 (104335)

13 1 and 12 (3229)

14 ((child* or teen* or adolescen* or p?ediatric* or infan* or neonat* or newborn* or minor*1) and (death or dying or terminal* ill* or end of life or incurable or life limiting)).ti. (2520)

15 ((child* or teen* or adolescen* or p?ediatric* or infan* or neonat* or newborn* or minor*1) adj3 (death or dying or terminal* ill* or end of life or incurable or life limiting)).ti,ab,id. (5478)

16 14 or 15 (6009)

17 13 and 16 (566)

18 8 or 17 (737)

19 exp palliative care/ or terminally ill patients/ or hospice/ or ("end of life" or palliative care or terminal care or terminal* ill* or hospice*).ti,ab,id. (22386)

20 (child* or teen* or adolescen* or p?ediatric* or infan* or neonat* or newborn* or minor*1).ti,ab,hw,id,jx. (951224)

21 19 and 20 (2491)

22 (canad* or british columbia or alberta or saskatchewan or manitoba or ontario or quebec or new brunswick or nouveau brunswick or nova scotia or prince edward island or newfoundland or labrador or nunavut or nwt or northwest territories or yukon).ab,ca,cq,hw,in,pl,ti. (292234)

23 21 and 22 (258)

24 18 or 23 (1017)

**EBSCOhost CINAHL Plus with Full-text via (1936 - November 4, 2020)**

S1 (MH "Attitude to Death") OR (MH "Death/PF") OR (MM "Death") OR TI(death or dying or "end of life" or euthanasia or MAID or "palliative care" or terminal*)

S2 (MH "Concept Formation") OR (MH "Perception") or TI (meaning or attitude* or perception* or belief* or perspective* or concept* or understand* or comprehen*)

S3 (MH "child development") or (MH "Child Psychology") or TI(child* or adolescen* or teen* or minor or minors or infan* or neonat* or newborn*) or SO(child* or adolescen* or teen* or minor or minors or infan* or neonat* or newborn*)

S4 S1 AND S2 AND S3

S5 (((child* or adolescen* or teen* or minor or minors or infan* or neonat* or newborn* ) n3 (meaning or attitude* or perception* or belief* or perspective* or concept* or understand* or comprehen*)) and ((meaning or attitude* or perception* or belief* or perspective* or concept* or understand* or comprehen*) n3 (death or dying "end of life" or euthanasia or MAID or "palliative care" or terminal*))

S6 S4 OR S5

S7 (MH "Palliative Care") OR (MH "Terminal Care+") OR (MH "Terminally Ill Patients+") OR (MH "Cancer Patients") OR "end of life" or "palliative care" or terminal* or hospice* or incurable or "life limiting" or "life shortening" or "impending death" or "active* dying" or dying w2 (child* or adolescen* or teen* or infan* or neonat* or newborn*)

S8 S6 AND S7

S9 (MH "Family Attitudes+") OR (MH "Family") or (MH "Caregivers") OR (MH "Caregiver Attitudes") OR (MH "Attitude of Health Personnel+") OR TI(family* or families* or parent* or brother* or sister* or sibling* or caregiver* or nurse* or physician* or oncologist* or cardiologist* or ((health* or "home care") n2 (worker* or professional*)))

S10 S1 AND S2 AND S9

S11 ( ((family* or families* or parent* or brother* or sister* or sibling* or caregiver* or nurse* or physician* or oncologist* or cardiologist* or ((health* or "home care") n2 (worker* or professional*))) n3 (meaning or attitude* or perception* or belief* or perspective* or concept* or understand* or comprehen*)) ) AND ( ((meaning or attitude* or perception* or belief* or perspective* or concept* or understand* or comprehen*) n3 (death or dying "end of life" or euthanasia or MAID or "palliative care" or "terminal care" or "terminal* ill*")) )

S12 S10 OR S11

S13 TI ( ((child* or teen* or adolescen* or pediatric* or paediatric* or infan* or neonat* or newborn*) and (death or dying or "terminal* ill*" or "end of life" or incurable or "life limiting" or "life shortening" or "active* dying")) ) OR AB ( ((child* or teen* or adolescen* or pediatric* or paediatric* or infan* or neonat* or newborn*) n3 (death or dying or "terminal* ill*" or "end of life" or incurable or "life limiting" or "life shortening" )) )

S14 S12 AND S13

S15 ( (MH "Palliative Care") OR (MH "Terminal Care+") OR (MH "Terminally Ill Patients+") OR (MH "Cancer Patients") OR "end of life" or "palliative care " or "terminal care" or "terminal* ill*" or hospice* ) AND ( canad* or "british columbia" or alberta or saskatchewan or manitoba or ontario or quebec or "new brunswick" or "nouveau brunswick" or "nova scotia" or "prince edward island" or newfoundland or labrador or nunavut or nwt or "northwest territories" or yukon ) AND ( child* or teen* or adolescen* or pediatric* or paediatric* or infan* or neonat* or newborn* )

S16 S8 OR S14 OR S15

**EBSCOhost Child Development & Adolescent Studies (Same strategy for Health Source: Nursing/Academic Edition, Philosopher's Index, Religion and Philosophy Collection, SocINDEX)**

S1 SU(death or thanatology) OR TI(death or dying or "end of life" or euthanasia or "assisted death" or MAID or "palliative care" or terminal*)

S2 SU(perception*) or SU("belief & doubt") OR TI(meaning or attitude* or perception* or belief* or perspective* or concept* or understand* or comprehen*)

S3 S1 AND S2

S4 SU("attitudes toward death")

S5 S3 OR S4

S6 SU("child development") or "child psychology" or " adolescent development" ) OR TI(child* or adolescen* or teen* or infan* or neonat* or newborn* or minor or minors)

S7 S5 AND S6

S8 SU("children & death") OR SU("teenagers & death")

S9 S2 AND S8

S10 (((child* or adolescen* or teen* or infan* or neonat* or newborn* or minor or minors) n3 (meaning or attitude* or perception* or belief* or perspective* or concept* or understand* or comprehen*)) and ((meaning or attitude* or perception* or belief* or perspective* or concept* or understand* or comprehen*) n3 (death or dying or afterlife or heaven or "end of life" or euthanasia or "assisted death" or MAID or "palliative care" or terminal*)))

S11 S7 OR S9 OR S10

S12 SU("palliative treatment") OR SU("terminal care") or SU("terminally ill children") or "end of life" or palliative or terminal* or hospice* or incurable or "life limiting" or "life shortening" or "impending death" or dying n2 (child* or adolescen* or teen* or infan* or neonat* or newborn*)

S13 S11 AND S12

S14 SU(family) OR SU(caregivers) OR SU("medical personnel") OR SU(physicians) OR SU(nurses) OR TI(family* or families* or parent* or brother* or sister* or sibling* or caregiver* or nurse* or physician* or oncologist* or cardiologist* or ((health* or "home care") n2 (worker* or professional*))

S15 S5 AND S14

S16 ( ((family* or families* or parent* or brother* or sister* or sibling* or caregiver* or nurse* or physician* or oncologist* or cardiologist* or ((health* or "home care") n2 (worker* or professional*))) n3 (meaning or attitude* or perception* or belief* or perspective* or concept* or understand* or comprehen*)) ) AND ( ((meaning or attitude* or perception* or belief* or perspective* or concept* or understand* or comprehen* ) n3 (death or dying or "end of life" or euthanasia or MAID or "palliative care" or "terminal care" or "terminal* ill*")) )

S17 S15 OR S16

S18 SU("children & death") OR SU("teenagers & death") or TI ( ((child* or teen* or adolescen* or pediatric* or paediatric* or infan* or neonat* or newborn*) and (death or dying or "terminal* ill*" or "end of life" or incurable or "life limiting" or "life shortening")) ) OR AB ( ((child* or teen* or adolescen* or pediatric* or paediatric* or infan* or neonat* or newborn*) n3 (death or dying or "terminal* ill*" or "end of life" or incurable or **or** "life shortening")) )

S19 S17 AND S18

S20 ( SU ("palliative treatment") OR SU("terminal care") or SU("terminally ill children") or OR "end of life" or palliative or "terminal care" or "terminal* ill*" or hospice* ) AND ( canad* or "british columbia" or alberta or saskatchewan or manitoba or ontario or quebec or "new brunswick" or "nouveau brunswick" or "nova scotia" or "prince edward island" or newfoundland or labrador or nunavut or nwt or "northwest territories" or yukon ) AND ( child* or teen* or adolescen* or pediatric* or paediatric* or infan* or neonat* or newborn* )

S21 S13 OR S19 OR S20

#### **Web of Science Core Collection: Citation Indexes (Advanced Search)**

#1 TS=(((child* or adolescen* or teen* or infan* or neonat* or newborn* or minor or minors) near/3 (meaning or attitude* or perception* or belief* or perspective* or concept* or understand* or comprehen*)) and ((meaning or attitude* or perception* or belief* or perspective* or concept* or understand* or comprehen*) near/3 (death or dying or "end of life" or euthanasia or "assisted death" or MAID or "palliative care" or terminal*))) AND TS=("end of life" or palliative or terminal* or hospice* or incurable or oncolog* or incurable or "life limiting" or "life shortening" or "impending death" or "active dying" or (dying near/2 (child* or adolescen* or teen* or infan* or neonat* or newborn*)))

#2 TS=(( family* or families* or parent* or brother* or sister* or sibling* or caregiver* or nurse* or physician* or oncologist* or cardiologist* or "healthcare worker*" or "home care worker") near/3 (meaning or attitude* or perception* or belief* or perspective* or concept* or understand* or comprehen*)) AND TS=( ((meaning or attitude* or perception* or belief* or perspective* or concept* or understand* or comprehen*) near/3 (death or "end of life" or euthanasia or MAID or "palliative care" or "terminal care" or "terminal* ill*")) ) AND TS=( ((child* or teen* or adolescen* or pediatric* or paediatric* or infan* or neonat* or newborn*) near/3 (death or dying or "terminal* ill*" or "end of life" or incurable or "life limiting" or "life shortening" or "active dying")) )

#3 TS=(("end of life" or "palliative care" or "terminal care" or "terminal* ill*" or hospice* ) AND ( canad* or "british columbia" or alberta or saskatchewan or manitoba or ontario or quebec or "new brunswick" or "nouveau brunswick" or "nova scotia" or "prince edward island" or newfoundland or labrador or nunavut or nwt or "northwest territories" or yukon ) AND ( child* or teen* or adolescen* or pediatric* or paediatric* or infan* or neonat* or newborn* ))

#4 #1 OR #2 OR #3

**Scopus**

TITLE-ABS-KEY(((child* or adolescen* or teen* or infan* or neonat* or newborn* or minor or minors) w/3 (meaning or attitude* or perception* or belief* or perspective* or concept* or understand* or comprehen*)) and ((meaning or attitude* or perception* or belief* or perspective* or concept* or understand* or comprehen*) w/3 (death or dying or "end of life" or euthanasia or MAID or "palliative care" or terminal*))) AND TITLE-ABS-KEY("end of life" or palliative or terminal* or hospice* or incurable or "life limiting" or "life shortening" or "impending death" or "active dying" or (dying w/2 (child* or adolescen* or teen* or infan* or neonat* or newborn*))) OR (TITLE-ABS-KEY(( family* or families* or parent* or brother* or sister* or sibling* or caregiver* or nurse* or physician* or oncologist* or cardiologist* or "healthcare worker*" or "home care worker") w/3 (meaning or attitude* or perception* or belief* or perspective* or concept* or understand* or comprehen*)) AND TITLE-ABS-KEY( ((meaning or attitude* or perception* or belief* or perspective* or concept* or understand* or comprehen* or fear or anxiety or anxious or scared) W/3 (death or dying or "end of life" or euthanasia or MAID or "palliative care" or "terminal care" or "terminal* ill*")) )) OR (TITLE-ABS-KEY(("end of life" or "palliative care" or "terminal care" or "terminal* ill*" or hospice*) AND ( canad* or "british columbia" or alberta or saskatchewan or manitoba or ontario or quebec or "new brunswick" or "nouveau brunswick" or "nova scotia" or "prince edward island" or newfoundland or labrador or nunavut or nwt or "northwest territories" or yukon ) AND ( child* or teen* or adolescen* or pediatric* or paediatric* or infan* or neonat* or newborn* )))

**ProQuest Dissertations & Theses Global and ProQuest Sociological Abstracts**

(((child* or adolescen* or teen* or infan* or neonat* or newborn* or minor or minors) near/3 (meaning or attitude* or perception* or belief* or perspective* or concept* or understand* or comprehen*)) and ((meaning or attitude* or perception* or belief* or perspective* or concept* or understand* or comprehen*) near/3 (death or dying or "end of life" or euthanasia or MAID or "palliative care" or terminal*))) AND ("end of life" or palliative or terminal* or hospice* or incurable or oncolog* or incurable or "life limiting" or "life shortening" or "impending death" or or "active dying" or (dying near/2 (child* or adolescen* or teen* or infan* or neonat* or newborn*)))

OR

(( family* or families* or parent* or brother* or sister* or sibling* or caregiver* or nurse* or physician* or oncologist* or cardiologist* or "healthcare worker*" or "home care worker") near/3 (meaning or attitude* or perception* or belief* or perspective* or concept* or understand* or comprehen*)) AND ( ((meaning or attitude* or perception* or belief* or perspective* or concept* or understand* or comprehen*) near/3 (death or dying or afterlife or heaven or "end of life" or euthanasia or MAID or "palliative care" or "terminal care" or "terminal* ill*")) ) AND ( ((child* or teen* or adolescen* or pediatric* or paediatric* or infan* or neonat* or newborn*) near/3 (death or dying or "terminal* ill*" or "end of life" or incurable or "life shortening" or "life limiting" or "active* dying")) )

OR

(("end of life" or palliative or "terminal care" or "terminal* ill*" or hospice* ) AND ( canad* or "british columbia" or alberta or saskatchewan or manitoba or ontario or quebec or "new brunswick" or "nouveau brunswick" or "nova scotia" or "prince edward island" or newfoundland or labrador or nunavut or nwt or "northwest territories" or yukon ) AND ( child* or teen* or adolescen* or pediatric* or paediatric* or infan* or neonat* or newborn* ))
